# Influence of *APOE* Genotype on Whole-Brain Functional Networks in Cognitively Normal Elderly

**DOI:** 10.1371/journal.pone.0083205

**Published:** 2013-12-11

**Authors:** Eun Hyun Seo, Dong Young Lee, Jong-Min Lee, Jun-Sung Park, Bo Kyung Sohn, Young Min Choe, Min Soo Byun, Hyo Jung Choi, Jong Inn Woo

**Affiliations:** 1 Department of Neuropsychiatry, Seoul National University Hospital, Seoul, Korea; 2 Department of Neuropsychiatry, Seoul National University College of Medicine, Seoul, Korea; 3 Interdisciplinary Program of Cognitive Science, Seoul National University, Seoul, Korea; 4 Department of Biomedical Engineering, Hanyang University, Seoul, Korea; Beijing Normal University,Beijing, China

## Abstract

This study aimed to investigate the influence of apolipoprotein E (APOE) ε4 allele on whole-brain functional networks in cognitively normal (CN) elderly by applying graph theoretical analysis to brain glucose metabolism. Eighty-six CN elderly [28 APOE ε4 carriers (ε4+) and 58 non-carriers (ε4-)] underwent clinical evaluation and resting [^18^F] fluorodeoxyglucose positron emission tomography scan. Whole-brain functional networks were constructed from correlations of the 90 regions of interest using the automated anatomical labeling template, and analyzed using graph theoretical approaches. The overall small-world property seen in ε4- was preserved in ε4+. However, both local clustering and path length were lower in ε4+ compared to ε4-. In terms of the hubs of functional networks, ε4+ showed decreased centrality of the right hippocampus but increased centrality of several brain regions associated with the default mode network compared to ε4-. Our results indicate that genetic vulnerability to Alzheimer’s disease may alter whole-brain functional networks even before clinical symptoms appear.

## Introduction

Alzheimer’s disease (AD) is the most common cause of dementia, and is characterized by progressive cognitive decline including memory impairment. The apolipoprotein E (APOE) ε4 allele is a major genetic risk factor for late-onset AD and its inheritance lowers the age at onset of AD [1,2]. Neuroimaging studies on the influence of APOE ε4 allele on brain function and structure in cognitively normal (CN) elderly individuals can provide important clues for understanding the preclinical AD process which occurs in the brain. 

The AD process is known to disrupt higher-order neural networks, as well molecular pathways, neuronal subpopulations, and local circuits in specific brain regions [3]. Evidence from various clinical and preclinical studies [4] supports the idea of AD being a disconnection syndrome implying that a network-based approach at the whole-brain level is critical to understand brain alterations and cognitive deficits associated with the AD process.

Graph theoretical analysis provides a mathematical and conceptual framework for understanding the brain as a whole network [5]. Graph theory allows capturing various aspects of the brain networks’ global topological organization as well as local contributions of each area to network function [6]. Many studies have confirmed that the human brain has a “small-world” property including a much higher local clustering and similar path length compared to matched random networks, which provides a balance between local specialization and global integration to maximize the efficiency of information processing [7–10]. Furthermore, as this approach primarily considers the integration of each region of the brain into a global unit rather than regarding each region as independent, it probably detects early subtle alterations with high sensitivity [9]. Nevertheless, whole-brain functional network properties based on the graph theoretical approach have not yet been explored in cognitively normal (CN) individuals with AD risk genes including the APOE ε4 allele. 

Mounting evidence suggests that AD begins with subtle alteration of synaptic function [11]. Regional cerebral glucose metabolism (rCMglc), measured by [^18^F] fluorodeoxyglucose positron emission tomography (FDG-PET), is a reliable and a highly sensitive index of synaptic function [12,13]. In line with this, clinical AD patients show the typical pattern of regional glucose hypometabolism. Even CN individuals with the genetic risk or family history of AD were reported to show decreased glucose metabolism in regions similar to those where AD patients show typical hypometabolism [14–17].

Therefore, we aimed to investigate the influence of APOEε4 allele on whole-brain functional networks in CN elderly people by applying graph theoretical analyses to the resting FDG-PET data.

## Materials and Methods

### Participants

In this study, 86 CN elderly, aged 55-84 years were included. They were recruited among community-dwelling elderly people who participated in a service program for the early detection and management of dementia at three centers located in Seoul, Korea (two public health centers and one the Dementia clinic of Seoul National University Hospital) from January 2007 to September 2011. Participants underwent clinical evaluation, APOE genotyping, and resting FDG-PET scan. None of the subjects had dementia or mild cognitive impairment, and they all had received a clinical dementia rating [18] score of 0. The exclusion criteria were presence of any serious medical, psychiatric, and neurological disorders that could affect the mental function; evidence of focal brain lesions on magnetic resonance imaging; presence of severe behavioral or communication problems that would make a clinical examination or FDG-PET scan difficult; left-handedness; absence of a reliable informant; and inability to read Korean. Individuals with minor physical abnormalities (e.g., diabetes with no serious complications, essential hypertension, mild hearing loss, or others) were included. The Institutional Review Board of the Seoul National University Hospital, Korea, approved the study protocol. All subjects gave written informed consent.

### Determination of the APOE Genotype

Genomic DNA was extracted from venous blood using standard procedures. APOE genotyping was performed according to the previously described method [19]. 

### Clinical and neuropsychological assessments

All subjects were examined by neuropsychiatrists, who had advanced training in neuropsychiatry and dementia research, according to the Consortium to Establish a Registry for Alzheimer’s Disease (CERAD) clinical and neuropsychological assessment batteries. Standard administration of the CERAD battery has been described previously in detail [20,21]. Reliable informants were interviewed to acquire accurate information regarding the cognitive, emotional, and functional changes as well as the subject’s medical history. A panel consisting of four psychiatrists with expertise in dementia research made the clinical decisions after reviewing all available raw data from clinical evaluations.

### PET Image acquisition and preprocessing

PET studies were performed using the ECAT EXACT 47 scanner (Siemens-CTI, Knoxville, Tenn., USA), which has an intrinsic resolution of 5.2-mm full width at half maximum (FWHM) and the images of 47 contiguous transverse planes with a 3.4-mm thickness for a longitudinal field of view of 16.2 cm. The mean interval between PET scan and clinical assessment was 10 days. All of the [^18^F] FDG PET scans were performed in a dimly lit room with minimal auditory stimulation during both the injection and PET scanning. Subjects were placed in the supine position with eyes closed during the scanning to minimize the confounding effects of any activity. More specific information on image acquisition procedures has been described previously in detail [22]. 

Imaging data were preprocessed using Statistical Parametric Mapping 2 (SPM2; Institute of Neurology, University College of London, UK) implemented in the Matlab (Mathworks Inc, USA). Before statistical analysis, all images were spatially normalized to the Montreal Neurological Institute (MNI; McGill University, Montreal, Canada) space. Normalized images were smoothed by convolution using an isotropic Gaussian kernel with 12 mm full width at half maximum. 

### Construction of functional networks using graph theoretical approaches

**Figure 1:**
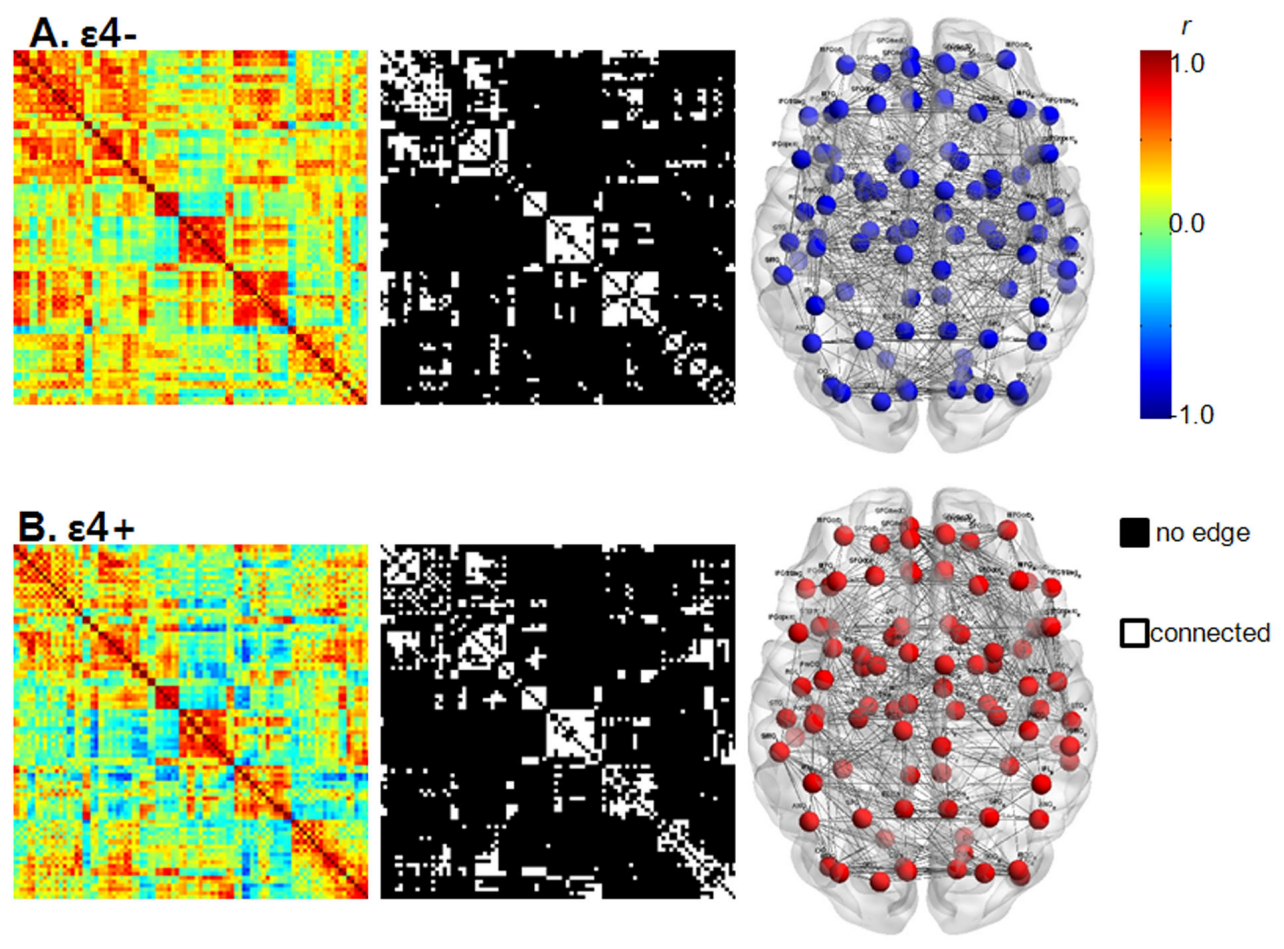
Whole-brain functional networks in APOE ε4- (A) and ε4+ (B). The first column displays the correlation matrices obtained from partial correlation coefficients (indicated by color bar, range from -1.0 to 1.0) between 90 regions of interests controlling for age, education and gender. The second column displays binary matrices thresholded at a fixed density of 15%. The third row illustrates the corresponding brain connectivity graph from the binary matrices. Graphs were visualized using the BrainNet viewer (NKLCNL, Beijing Normal University).

In graph theory, a network is a set of nodes and edges between pairs of nodes [6]. In our study, nodes were represented by 90 regions of interest (ROI) defined using automated anatomical labeling (AAL) template [23], which has been broadly used in brain network studies [7,24–26]. In the AAL template, 45 ROIs in each hemisphere except the cerebellum were defined based on an anatomical parcellation of MNI-normalized single-subject high-resolution T1 volume [23]. The whole-brain functional networks were constructed with these 90 cortical and subcortical mean values of rCMglc (Table S1 in the informationS 1). The rCMglc of each ROI was globally normalized with respect to the mean metabolic rate for glucose in each individual’s whole brain. We selected the global normalization procedure because it has a higher signal-to-noise ratio compared to the cerebellar count normalization method [27] and correlation coefficients are obtained separately for each diagnostic group. Interregional correlation matrix (90X90) was acquired by partial correlation analysis controlling for the effects of age, gender, and education These partial correlation coefficients between every pair of ROIs represent edges (i.e., functional connections between nodes). To avoid complicated statistical descriptions in the following network analysis, our graph theoretical analysis was confined to a simple undirected and unweighted binary matrix (Figure 1). The interregional correlation matrix was then transformed into a binary matrix using the fixed density threshold method. Density is the fraction of present connections to possible connections [6]. Fixed density threshold method ensures that the graphs from two study groups have the same number of edges [28]. As there is no gold standard for a single threshold, we applied a wide range of density (D) (i.e., 10%≤ *D* ≤ 40%) with an incremental interval of 1% and repeated the full analysis for each density. This range of density was selected to estimate the small-world property as suggested in previous studies [28,29].

### Network analysis

Network parameters used in the current study are listed with mathematical definitions in Table S2 of the Supporting information S1. Two fundamental network parameters are the clustering coefficient and the characteristic path length. Clustering coefficient *C*_*i*_ of node *i* indicates the likelihood of the neighboring nodes to be connected to each other. Clustering coefficient for a network (*C*_*p*_ ) is the average *C*_*i*_ from entire nodes in the network, which quantifies the extent of local interconnectivity of information transfer in a network [6,30]. Characteristic path length (*L*_*p*_) is the mean minimum number of edges of the shortest path connecting any two nodes in a network, which quantifies the extent of functional integration of a network [6,30]. The distinctive combination of high *C*_*p*_ with short *L*_*p*_ is the key property of small-world network [30]. Matched random networks with the same node degree distribution as the brain networks in the present study were generated 1000 times repeatedly and the mean value of clustering coefficients (*C*_*p*_^*rand*^) and characteristic path length (*L*_*p*_^*rand*^) were used as representative parameters of random network. A network is considered as a small-world network if it shows much a higher *C*_*p*_ (γ = *C*_*p*_^real^/*C*_*p*_^rand^ ≫1) while a similar *L*_*p*_ (λ = L_*p*_^real^/*L*_*p*_^rand^1) in comparison with the matched random network [30]. That is, the small-world index σ = γ/λ is greater than 1. Small-worldness tests were performed repeatedly over a range of density (i.e., 10%≤ *D* ≤ 40%). 

For the local nodal characteristics, we employed betweenness centrality. Betweenness centrality, *B*_*i*_ of a node *i* is defined as the number of shortest paths between any two nodes that run through the node *i*, which quantifies how much information might traverse through the node, presuming that optimal paths are used [6,31]. *B*_*i*_ of a node *i* was used to determine candidate hubs in a network. The *B*_*i*_ was normalized as *b*_*i*_= *B*_*i*_ /averaged *B*_*i*_ for all nodes of the entire network. The nodes that have a high *b*_*i*_ (>1.5) are considered as functional hubs of a network. The *b*_*i*_ of each node was calculated at a fixed density of 15%. Certain density threshold, which ensures that all of the 90 ROIs are included and false-positive paths are minimized, was determined as a fixed density. The lowest density where the largest component size was 90 (i.e., all connected nodes included) was density of 15% in the present study. 

Calculations of these network parameters were performed using ‘Brain connectivity analysis software’ (http://www.brain-connectivity-toolbox.net) [6] and the MatlabBGL package (http://www.stanford.edu/~dgleich/progrma/matlab_bgl/).

### Statistical analyses

Differences in rCMglc between groups were measured on a voxel-by-voxel basis using the 2-sample t test (*p* < 0.001, uncorrected for multiple comparisons). Between-group differences in network parameters (*C*_*p,*_ *L*_*p,*_ and *b*_*i*_) were tested using a nonparametric permutation test with 5000 repetitions and the 95 percentile scores of each difference-distribution were considered as the critical values (*p*<0.05, one-tailed) [28,32]. To test if the observed group difference occurred by chance (the null hypothesis), we randomly reassigned each participant’s 90 ROI rCMglc values to one of the two groups. After the randomization procedure, the interregional correlation matrix was calculated again and a set of binary matrices was also obtained over the same density threshold range as in the real brain networks. *C*_*p,*_ *L*_*p,*_ σ, and *b*_*i*_ were calculated in each network separately, and then between-group differences in the network parameters were obtained. This procedure was repeated 5000 times and the 95 percentile scores of each difference-distribution were considered as the critical values (*p*<0.05, one-tailed). This nonparametric permutation test procedure was performed repeatedly at 10%≤*D*≤40%. We did not make any adjustment for multiple comparisons because we tried to explore the general trends of between-group differences through the wide range of density level rather than putting a separate and specific interpretation on the result at a certain density level.

## Results

### Subject characteristics

Demographic and neuropsychological characteristics of all the subjects are summarized in Table 1.Twenty-eight subjects were ε4+ (all carried the ε3/ε4 allele) and 58 were ε4- (8 subjects carried the ε2/ε3 allele and 50 subjects carried the ε3/ε3 allele). There were no significant differences between groups in age, gender, education, and eight cognitive test scores.

**Table 1:** Demographic characteristics of the subjects.

	ε4- (*n*=58)	ε4+ (*n*=28)
Age (SD), years	70.8 (6.8)	69.1 (4.7)
Education (SD), years	8.9 (5.2)	9.3 (4.7)
Gender (M/F)	22/36	7/21
MMSE score (SD)	26.5 (2.5)	26.4 (2.1)
Neuropsychological test		
Animal fluency	16.4 (4.7)	16.2 (4.7)
Boston naming	11.8 (2.2)	11.7 (2.3)
Word list learning	19.2 (4.5)	18.3 (4.1)
Word list recall	7.0 (1.9)	6.7 (1.9)
Word list recognition	9.4 (1.0)	9.3 (0.9)
Constructional praxis	10.3 (1.1)	10.1 (1.3)
Constructional recall	6.7 (2.9)	7.6 (2.6)

Values are given as mean (standard deviation) except for gender ε4- = cognitively normal elderly without apolipoprotein E ε 4 allele; ε4+ =cognitively normal elderly with apolipoprotein E ε 4 allele; MMSE= Mini mental status examination

### Comparison of rCMglc

**Figure 2:**
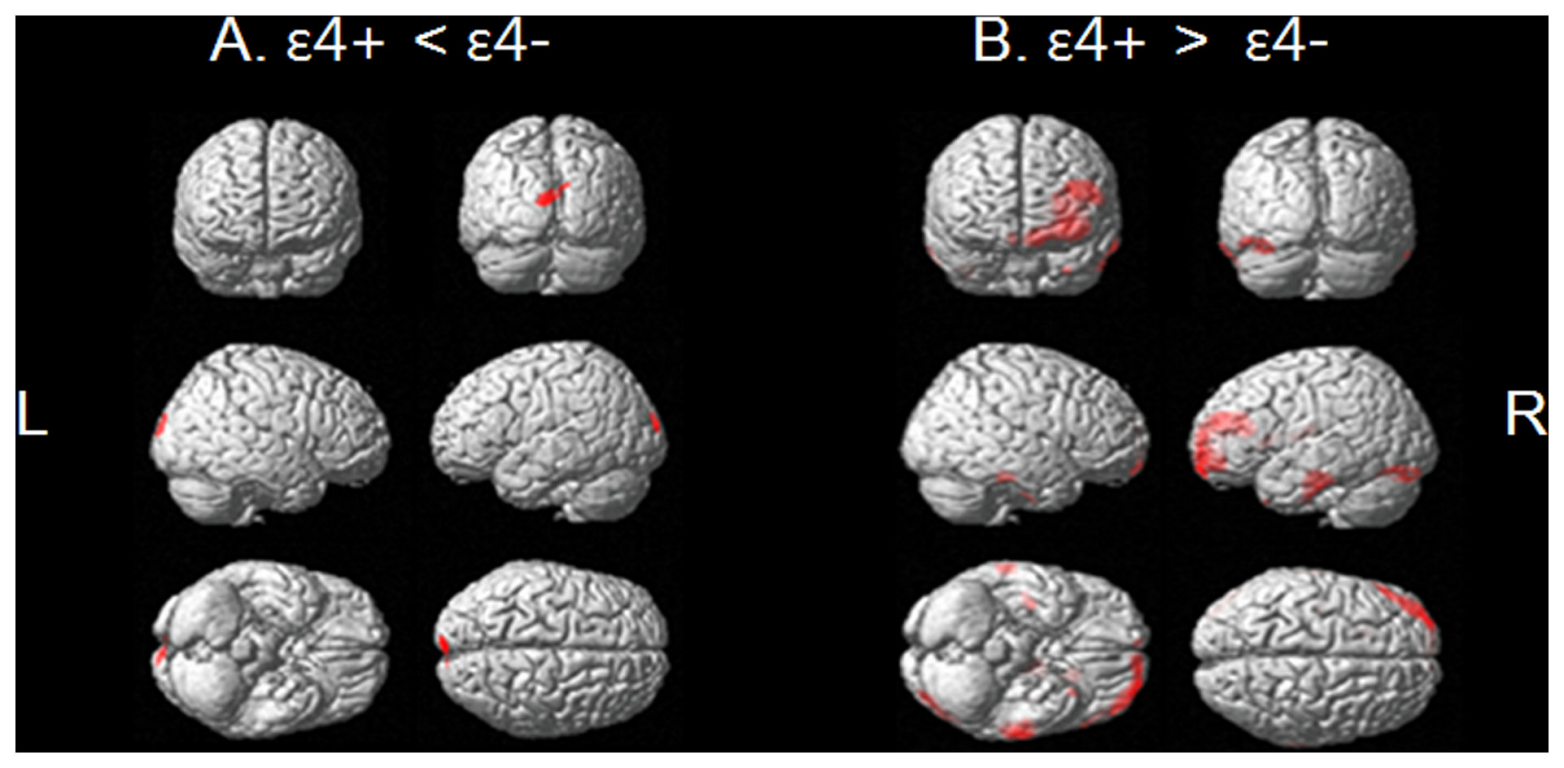
Brain areas showing regional glucose metabolism reductions in (A) ε4+ as compared with ε4-, and (B) in ε4- as compared with ε4+ (p < 0.001, uncorrected for multiple comparison) ε4+ = cognitively normal elderly with apolipoprotein E ε4 allele, ε4- = cognitively normal elderly without apolipoprotein E ε4 allele. L = left, R = right.

Compared to ε4-, ε4+ showed a significant decrease in rCMglc in the bilateral cuneus, whereas ε4+ showed an increase in rCMglc in the left frontal regions and bilateral temporal gyri (Figure 2 and Table 2). 

**Table 2:** Brain areas showing significant regional glucose metabolism differences between ε4+and ε4-*.

Brain region (BA)	MNI coordinates			z-score		Cluster size	
		x	y	z			
**Decreased metabolism in ε4+ compared to ε4-**							
	L cuneus (18)	-4	-100	16	3.41		43
	R cuneus (19)	12	-96	26	3.22		
	**Increased metabolism in ε4+ compared to ε4-**						
	L superior frontal gyrus (10)	-30	64	-8	5.13		1008
	L medial frontal gyrus (11)	-12	66	-16	4.63		
	L middle frontal gyrus (11)	-38	56	-12	4.59		
	L fusiform gyrus (20)	-60	-22	-36	4.24		304
	L inferior temporal gyrus (20)	-68	-18	-22	3.77		
	L thalamus (-)	-12	-10	12	4.24		247
	R inferior temporal gyrus (20)	70	-30	-28	3.96		97
	L inferior occipital gyrus (18)	-40	-86	-20	3.90		196
	R uncus (20)	38	-14	-40	3.51		47
	L caudate nucleus (-)	-18	18	6	3.49		182
	L superior temporal gyrus (38)	-30	18	-42	3.36		22
	L insula (13)	-38	-14	18	3.35		38

*p < 0.001, uncorrected

ε4- = cognitively normal elderly without apolipoprotein E ε 4 allele; ε4+ =cognitively normal elderly with apolipoprotein E ε 4 allele; BA= brodmann areas; L = left; R = Right

### Global network properties and their alterations in APOE ε4 carriers

**Figure 3:**
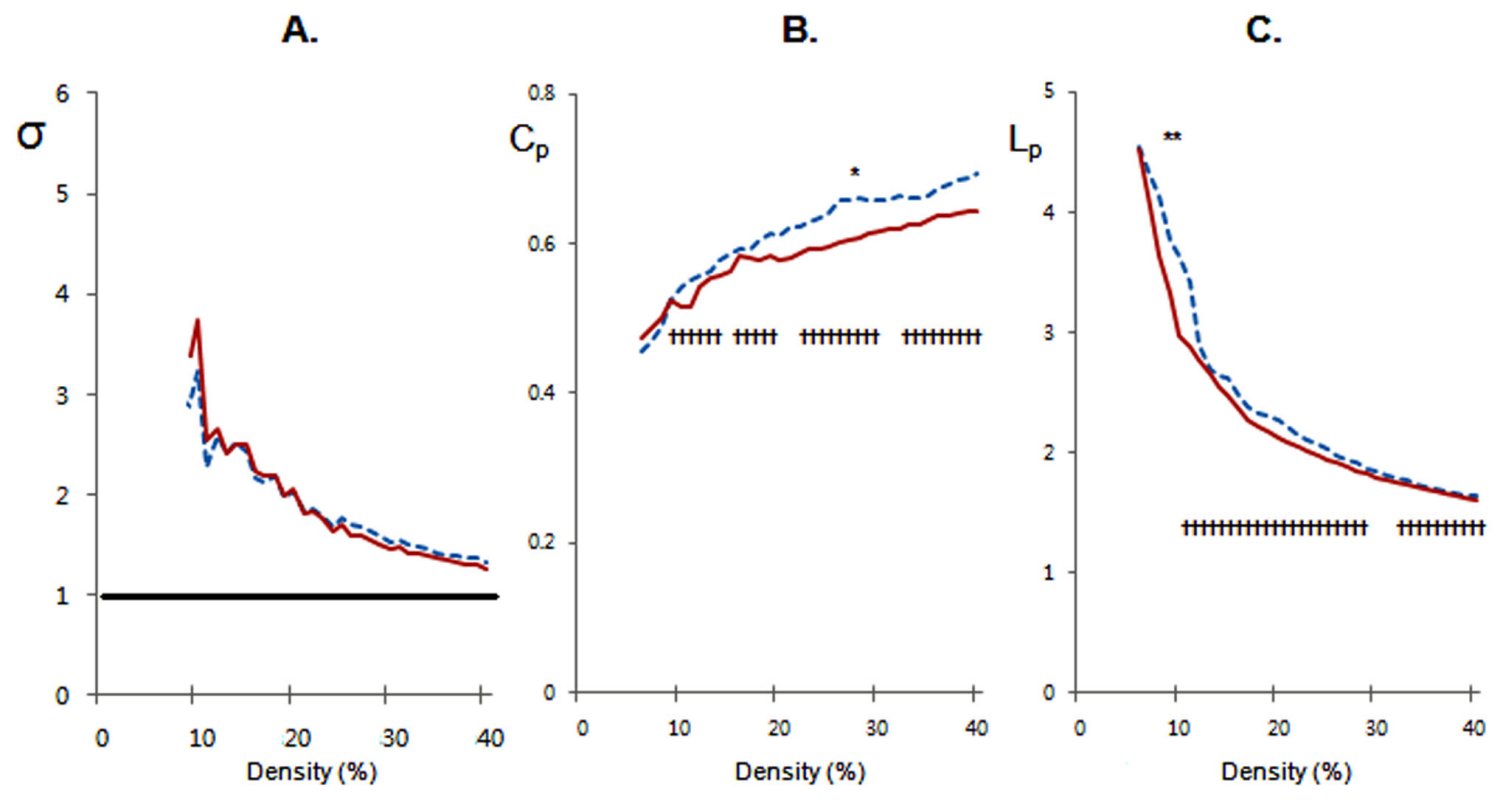
APOE ε4-related alterations in small-world parameters in cognitively normal elderly. (**A**) Small-world index (σ) from APOE ε4 carriers (ε4+) and APOE ε4 non-carriers (ε4-). (**B**) The clustering coefficients (C_p_).^*^ *p*<0.05 (density 28%); ^†^ *p*<0.06 (density 10%~14%, 16%~20%, 22%~30%, and 32%~40%). (**C**) The characteristic path length (L_p_). *p<0.05 (density 10% and 11%); ^†^ *p*<0.06 (density 8%~30% and 32%~40%). ε4- = blue dotted line; ε4+ = red solid line.

Both groups demonstrated small-world property (σ >1) over an entire range of density (Figure 3A). Between-group comparison revealed that σ was not significantly different. On the other hand, in terms of individual parameters, ε4+ showed a significantly lower *C*_*p*_ than ε4- at a density of 28%, and a non-significant trend toward lower *C*_*p*_ over a wide range of density (Figure 3B). ε4+ also showed a significantly shorter *L*_*p*_ than ε4- at a density of 10% and 11%, and a non-significant trend toward shorter *L*_*p*_ over a wide range of density (Figure 3C).

### Functional hubs and their alterations in APOE ε4 carriers

**Figure 4:**
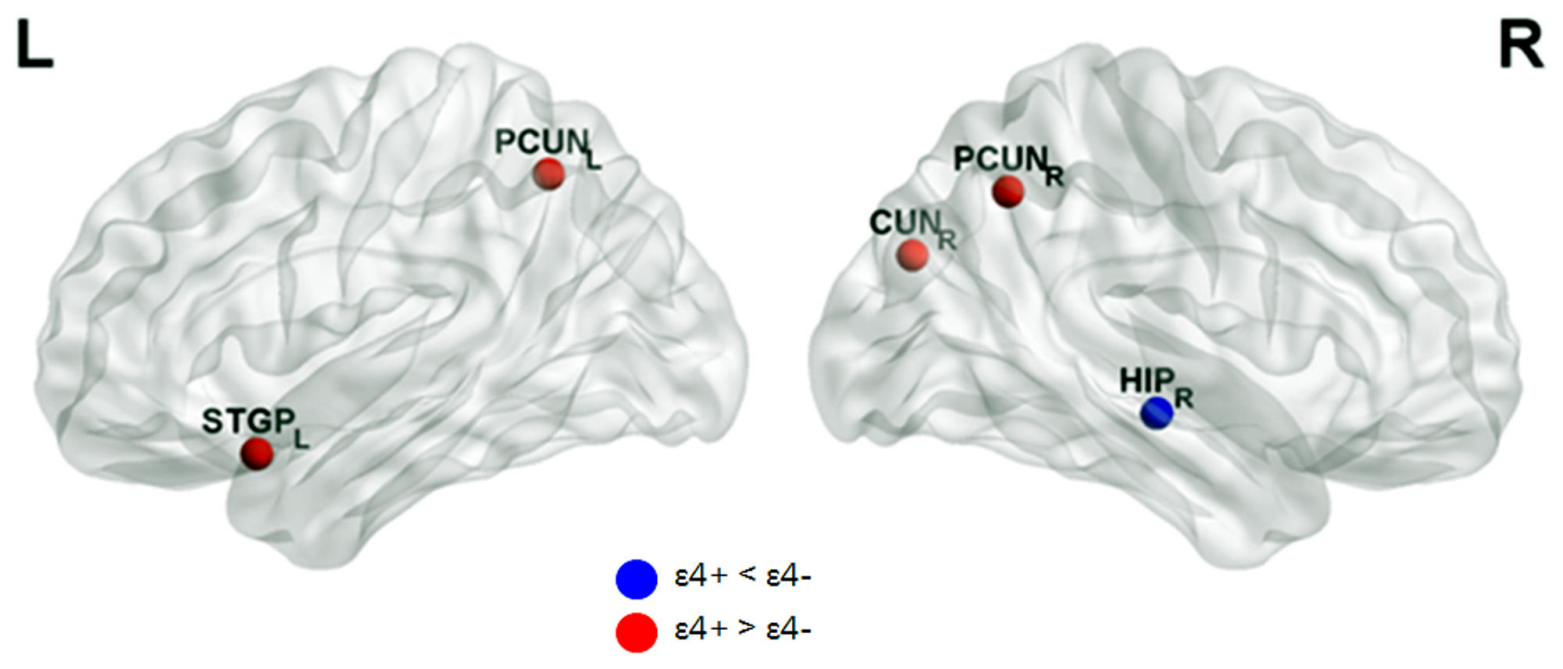
APOEε4-related alterations in hub regions. Hubs were visualized using BrainNet Viewer package (NKLCNL, Beijing Normal University). Blue circle indicates the regions where APOE ε4 carriers (ε4+) showed decreased betweenness centrality (b_i_) compared with APOE ε4 non-carriers (ε4-). Red circle indicates the regions where ε4+ showed increased *b*_*i*_ compared with ε4-.

The number of identified hubs was reduced in ε4+ compared with ε4- (26 hubs for ε4- vs. 17 hubs for ε4+). A majority of hubs were identified in the multimodal or unimodal association cortex in both groups. However, specific hub regions were dissimilar. In ε4-, hubs were located mainly in the frontal and temporal lobes, such as the bilateral inferior frontal regions, bilateral middle frontal gyri, right hippocampus, right parahippocampal gyrus and bilateral temporal gyri. On the other hand, in ε4+, hubs were located mainly in the posterior regions and the cingulate regions, such as the bilateral precuneus, right cuneus, bilateral middle cingulate gyri and right anterior cingulate (Tables S3 and S4). Further statistical analysis revealed that ε4+ showed a significant decrease in *b*_*i*_ in the right hippocampus than ε4- (*b*_*i*_ =0.14 for ε4+, *b*_*i*_ = 2.23 for ε4-). In contrast, ε4+ showed a significant increase in *b*_*i*_ in the bilateral precuneus, left superior temporal pole, and right cuneus (Figure 4).

## Discussion

We investigated the influence of *APOE* ε4 allele on whole-brain functional networks in CN elderly using graph theoretical analysis of FDG-PET data. Our study revealed that both local clustering and path length were lower in APOE ε4+ CN elderly than in ε4- CN elderly, while the overall small-world property seen in ε4- CN elderly was preserved in ε4+ CN elderly. Additionally, ε4+ CN elderly also showed regional alterations in functional hubs compared to ε4- CN elderly. 

To date, whole-brain functional network properties based on the graph theoretical approach have not been explored in CN individuals with AD risk genes including the APOE ε4 allele. Disorganization of network topology (i.e., a lower local clustering with a shorter path length) can be a characteristic for CN elderly with APOE ε4 allele. In a complex network, a random network has a lower clustering coefficient and a shorter path length, while a regular network has a greater clustering coefficient and a longer path length. Therefore, our results indicate that the APOE ε4 allele-associated alteration in global network topology tends toward increasing randomness, resulting in less efficient information flow in the brain, which is generally associated with brain disease [33].

Local clustering reflects the degree of closeness between neighboring brain regions. Although negative cognitive consequences were not apparent in the current study, information flow between local neighboring areas in whole-brain functional networks seemed to be less efficient in ε4+. The result of decreased local clustering in ε4+ CN elderly is compatible with the findings from previous functional network studies based on the graph theoretical approach for mild cognitive impairment (MCI) and AD [25,34–36] thereby suggesting that the presence of ε4 allele can alter the topology of whole-brain functional networks even before the clinical symptoms of AD appear. Decreased local clustering in ε4+ CN elderly may be partly explained by the patterns of regional alteration in brain metabolism. The ε4+ CN elderly not only showed localized hypometabolism typically in the posterior regions, but also focal hypermetabolism in the frontal and temporal regions. As the local clustering (*C*_*p*_,) of network is based on the correlation between adjacent brain regions, ε4+ CN elderly with such alteration patterns of regional metabolism showed lower local clustering than ε4- CN elderly. On the other hand, path length in ε4+ was shorter, implying that more long-range functional connections may be recruited in ε4+ CN elderly compared to ε4- CN elderly. In combination with the result of ε4+-associated hypermetabolism in frontal and temporal regions, it seems reasonable to hypothesize that shorter path length in ε4+ is a compensatory recruitment for early local pathology (e.g., decreased local clustering). 

In terms of local properties, ε4+ showed significantly reduced centrality of the right hippocampus compared to ε4-. Negative effect of the APOEε4 gene on the hippocampus in healthy samples has been observed in many of structural and functional neuroimaging studies [37–39]. Although the ε4+ group displayed intact memory performance, the key area related to episodic memory appeared to be less effective as hubs in ε4+ than in ε4-. In contrast, there were other brain regions showing increased centrality in ε4+ compared to ε4- (bilateral precuneus, right cuneus, left superior and middle temporal pole, left posterior cingulate gyrus). These regions generally correspond to a part of the default mode network (DMN). The APOE effect has been proposed as an example of antagonistic pleiotropy, in which APOE ε4 offers benefits during early and middle age, and causes maximal compensatory recruitment by invoking additional brain regions to maintain cognitive performance in old age, but once the burden of AD increases, compensatory processes cannot sustain premorbid cognitive function levels [40,41]. In fact, MCI and AD show reduced functional connectivity in these areas [42–44]. Taken together, our finding of ε4-related increase in centrality within the DMN could be speculated as a compensatory process to maintain a comparable level of cognitive performance. Relevant to our finding, recent resting-state functional magnetic resonance imaging (fMRI) studies demonstrated increased DMN functional connectivity in young or non-demented ε4+ individuals [45–47]. Task fMRI studies of nondemented elderly with ε4+ also showed increased activation in AD-associated regions while achieving normal level of memory performance[37,48].

In the current study, ε4+ CN elderly showed hypermetabolism in the frontal and temporal regions, as well as hypometabolism in the bilateral cuneus, while previous FDG-PET studies assessing the effect of ε4 gene in CN elderly reported only hypometabolism in the brain regions similar to the AD-affected areas [17,49]. This discrepancy between our results and other results appears to be related to the differences in characteristics of subjects, such as family history (FHx) of AD and age. Most previous studies included only FHx positive subjects, whereas we did not include FHx of AD as an inclusion or exclusion criteria and only 23.5% of subjects had a FHx of AD. Synergic negative influence of FHx of AD on the effect of expression of ε4 on brain function, as observed in an fMRI study [44], may explain the discrepancy between our results and other results. Age of subjects may also contribute to this discrepancy. The effect of APOE ε4 on the risk of AD increases steadily between ages 40 and 60 years but declines with age thereafter [50]. We included only elderly participants, whereas all previous studies included a large number of middle-aged people.

There are several limitations and future directions that need to be addressed. First, the sample sizes of two study groups were uneven and that of the ε4+ group was relatively small (*n*=28) considering the number of ROIs. In addition, 8 among 58 ε4- subjects carried ε2 allele, known to lower AD risk, while no ε4+ subjects have ε2 allele. Therefore, the presence of ε2 allele may affect the difference between ε4+ and ε4- group. It is needed that future studies with a larger sample size and balanced sample characteristics between ε4 carrier and non-carrier group replicate our results to confirm APOE ε4-related alterations in network property. Second, a cross-sectional design was used in the current study. Based on the present findings, the diagnostic or prognostic value of network parameters needs to be further explored in a longitudinal study. Third, this study did not investigate whether the ε4-related findings in brain network were mediated by amyloid-beta (Aβ) burden, although ε4 allele is known to affect Aβ deposition as well as brain glucose metabolism [51]. Further investigation of this issue will help to clarify the underlying mechanism of whole-brain functional network alterations in CN elderly with AD risk gene. Finally, we used AAL atlas which consists of 90 anatomical nodes. Although the small-world property was robust regardless of the parcellation method [24,52], different network definitions by using other parcellation templates (e.g., higher-resolution or functional template) might lead to different implications. 

In conclusion, our results for global functional network properties based on the graph theoretical approach indicate that although the overall small-world property is preserved in ε4+ CN elderly, as well as in ε4- CN elderly, individual characteristics of the network parameters are quite different in ε4+ compared to ε4-. Specifically, functional relatedness between neighboring brain regions reflected in local clustering coefficient appears to be decreased, and more long-range functional connections may be recruited as a possible compensation for decreased local clustering in ε4+ CN elderly. Overall, our results suggest that the APOE ε4-related genetic vulnerability for AD may alter whole-brain functional networks even before the clinical symptoms of AD appear. 

## Supporting Information

Information S1 **The following files are included in Information S1 File:** **Table S1.** Anatomical parcellation defined by automated anatomical labeling atlas and abbreviations for the region. **Table S2.** Mathematical definitions of network parameters used in the study [6]. **Table S3.** Functional hubs and node characteristics in ε4-. **Table S4.** Functional hubs and node characteristics in ε4+.
(DOC)
