# Supplementary material for: Influence of APOE Genotype on Whole-Brain Functional Networks in Cognitively Normal Elderly
Source: PLoS One. 2013 Dec 11;8(12):e83205. doi: 10.1371/journal.pone.0083205 (PMC3859658; doi:10.1371/journal.pone.0083205)
Supplement: Information S1 — The following files are included in Information S1 File: Table S1. Anatomical parcellation defined by automated anatomical labeling atlas and abbreviations for the region. Table S2. Mathematical definitions of network parameters used in the study [6]. Table S3. Functional hubs and node characteristics in ε4-. Table S4. Functional hubs and node characteristics in ε4+. (DOC) [file pone.0083205.s001.doc]

**Supporting information**

Table S1. Anatomical parcellation defined by automated anatomical labeling atlas and abbreviations for the regions

| **Abbreviations** | **Regions** |
| --- | --- |
| PreCG | Precental gyrus |
| SFGdor | Superior frontal gyrus, dorsolateral |
| SFGorb | Superior frontal gyrus, orbital part |
| MFG | Middle frontal gyrus |
| MFGorb | Middle frontal gyrus, orbital part |
| IFGoperc | Inferior frontal gyrus, opercular part |
| IFGtriang | Inferior frontal gyrus, triangular part |
| IFGorb | Inferior frontal gyrus, orbital part |
| ROL | Rolandic operculum |
| SMA | Supplementary motor area |
| OLF | Olfactory cortex |
| SFGmed | Superior frontal gyrus, medial |
| SFGmedO | Superior frontal gyrus, medial orbital |
| REC | Gyrus rectus |
| INS | Insula |
| ACC | Anterior cingulate and paracingulate gyri |
| MCC | Median cingulate and paracingulate gyri |
| PCC | Posterior cingulate gyrus |
| HIP | Hippocampus |
| PHG | Parahippocampal gyrus |
| AMYG | Amygdala |
| CAL | Calcarine fissure and surrounding cortex |
| CUN | Cuneus |
| LING | Lingual gyrus |
| SOG | Superior occipital gyrus |
| MOG | Middle occipital gyrus |
| IOG | Inferior occipital gyrus |
| FFG | Fusiform gyrus |
| PoCG | Postcentral gyrus |
| SPG | Superior parietal gyrus |
| IPL | Inferior parietal, but supramarginal and angular gyri |
| SMG | Supramarginal gyrus |
| ANG | Angular gyrus |
| PCUN | Precuneus |
| PCL | Paracentral lobule |
| CAU | Caudate nucleus |
| PUT | Lenticular nucleus, putamen |
| PAL | Lenticular nucleus, pallidum |
| THA | Thalamus |
| HES | Heschl gyrus |
| STG | Superior temporal gyrus |
| STGP | Temporal pole: superior temporal gyrus |
| MTG | Middle temporal gyrus |
| MTGP | Temporal pole: middle temporal gyrus |
| ITG | Inferior temporal gyrus |

| Table S2. Mathematical definitions of network parameters used in the study | | |
| --- | --- | --- |
| Parameter | Definitions | Mathematical expression |
| Degree, *k_i_* | The number of connections that link it to the rest of the network | | where 𝑁 is the set of all nodes in the network, 𝑎𝑖𝑗 is the connection status between 𝑖 and 𝑗. When an edge exists between these two nodes, 𝑎𝑖𝑗=1, otherwise 𝑎𝑖𝑗 =0   |  | | --- | |  | | | --- | --- | --- | |  | |
| Clustering Coefficients, *C_p_* | the average *C_i_* from entire nodes in the network, where *C_i_* is the likelihood that neighbors of a node will also be connected to each other | where *n* is the number of nodes, *t_i_* is number of triangles around node *i* and *C_i_* is Clustering Coefficients of node i (*C_i_* =0 for *k_i_* <2) |
| Characteristic path length, *L_p_* | the mean minimum number of edges of the shortest path connecting any two nodes |  |
|  |  | Where *d_ij_* is shortest path length (distance) between 𝑖 and 𝑗 and 𝐿𝑖 is the average distance between node 𝑖 and all other nodes |
| Small-worldness, σ  Betweenness centrality, *B_i_* | A network is considered as a small-world network if it show much higher *C_p_* while similar *L_p_* in comparison with the matched random networks  the number of shortest paths between any two nodes that run through node *_i_* | | 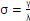 where γ=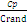, λ=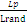,   | *C_p_* and 𝐶_rand_ are the clustering coefficients, and *L_p_* and 𝐿_rand_ are the characteristic path lengths of tested network and a random network respectively. Small-world networks often have  σ ≫1. | | --- | |  | | | --- | --- | --- | |  |   where *ρ_h_*_𝑗_ is the number of shortest paths between *h* and *j*, and 𝜌*_h_*_𝑗_(𝑖) is the number of shortest paths between *h* and *j* that pass through *i.* |
|  |  |  |

| Table S3. Functional hubs and node characteristics in ε4- | | | |
| --- | --- | --- | --- |
| **ROIs** | ***b_i_*** | **degree** | **regional *C_i_*** |
| Right Insula | 6.59 | 23 | 0.32 |
| Right inferior orbito frontal gyrus | 3.53 | 25 | 0.43 |
| Left Insula | 3.50 | 16 | 0.50 |
| Right superior temporal gyrus | 3.37 | 20 | 0.47 |
| Right middle temporal gyrus | 3.03 | 10 | 0.44 |
| Left inferior frontal opercular part | 2.84 | 22 | 0.48 |
| Left thalamus | 2.62 | 4 | 0.17 |
| Left middle temporal gyrus | 2.62 | 10 | 0.47 |
| Right inferior temporal gryus | 2.60 | 9 | 0.31 |
| Left precentral gyrus | 2.58 | 26 | 0.51 |
| Right middle frontal gyrus | 2.28 | 26 | 0.44 |
| Left inferior frontal triangular part | 2.25 | 22 | 0.47 |
| Right hippocampus | 2.23 | 9 | 0.47 |
| Right middle occipital gryus | 2.12 | 13 | 0.68 |
| Right supramarginal gryus | 2.11 | 18 | 0.53 |
| Left postcentral gyrus | 2.04 | 24 | 0.55 |
| Right parahippocampal gryus | 1.94 | 11 | 0.40 |
| Right angular gyrus | 1.92 | 22 | 0.52 |
| Right inferior frontal opercular part | 1.88 | 21 | 0.47 |
| Right superior temporal pole | 1.86 | 6 | 0.27 |
| Right middle temporal pole | 1.83 | 7 | 0.29 |
| Left inferior orbito frontal gyrus | 1.80 | 10 | 0.49 |
| Left middle occipital gryus | 1.72 | 14 | 0.64 |
| Right precentral gyrus | 1.62 | 27 | 0.54 |
| Left middle frontal gyrus | 1.59 | 23 | 0.49 |
| Right inferior frontal triangular part | 1.50 | 20 | 0.48 |

*Note*. Normalized betweenness centrality, *b_i_* >1.5 were listed as hubs in a descending order

*C_i_* denotes regional clustering coefficient of node i.

| Table S4. Functional hubs and node characteristics in ε4+ | | | |
| --- | --- | --- | --- |
| **ROIs** | ***b_i_*** | **degree** | **regional *C_i_*** |
| Right superior temporal gyrus | 5.47 | 28 | 0.38 |
| Left middle temporal gryus | 4.25 | 17 | 0.47 |
| Right insula | 3.16 | 26 | 0.41 |
| Right cuneus | 3.07 | 19 | 0.50 |
| Left precuneus | 3.04 | 18 | 0.43 |
| Right precuneus | 2.53 | 18 | 0.47 |
| Right middle temporal pole | 2.51 | 21 | 0.47 |
| Left insula | 2.41 | 22 | 0.42 |
| Right middle cingulate | 2.36 | 12 | 0.50 |
| Right anterior cingulate | 2.26 | 19 | 0.47 |
| Right superior frontal gyrus | 2.15 | 18 | 0.46 |
| Left supramarginal gyrus | 2.07 | 10 | 0.38 |
| Left middle cingulate | 2.05 | 12 | 0.52 |
| Right inferior orbito frontal gyrus | 2.02 | 24 | 0.50 |
| Right precentral gyrus | 1.86 | 14 | 0.44 |
| Left superior temporal pole | 1.74 | 25 | 0.46 |
| Right frontal triangular part | 1.61 | 19 | 0.51 |

*Note*. Normalized betweeness centrality, *b_i_* >1.5 were listed as hubs in a descending order

*C_i_* denotes regional clustering coefficient of node i.
